# Prolonged sedation in ARDS patients with inhaled anesthetics: our experience

**DOI:** 10.1186/cc12324

**Published:** 2013-03-19

**Authors:** S Redaelli, P Mangili, V Ormas, S Sosio, L Peluso, F Ponzoni, N Patroniti, A Pesenti

**Affiliations:** 1 University of Milan-Bicocca, Monza, Italy

## Introduction

We report our experience in the use of isoflurane for prolonged sedation in severe ARDS patients. Prolonged sedation in the ICU may be difficult because of tolerance, drug dependence and withdrawal, drug interactions and side effects. Inhaled anesthetics have been proposed for sedation in ventilator-dependent ICU patients. AnaConDa is a device that allows a safety and easy administration of inhaled anesthetics in the ICU.

## Methods

From January 2009 to June 2012, 15 patients were sedated with isoflurane by means of the AnaConDa device. We consider administration of isoflurane as a washout period from common sedative drugs in patients with (at least one of): high sedative drug dosage (propofol ≥300 mg/hour or midazolam ≥8 mg/hour) to reach the target Richmond Agitation Sedation Score (RASS) or inadequate paralysis; two or more hypnotic drugs to reach the target RASS (propofol, midazolam, hydroxyzine, haloperidol, diazepam, quetiapine); and hypertriglyceridemia. During isoflurane administration previous hypnotic drugs were interrupted. We retrospectively collected data before, during and after administration of isoflurane: hemodynamic parameters, renal and hepatic function, level of sedation (RASS) and sedative drug dosage. All data are reported as mean ± standard deviation, otherwise as median (minimum to maximum).

## Results

Mean age was 43 ± 12 years and SAPS II was 35.7 ± 11; 13 patients were treated with ECMO for severe ARDS and four had a history of drug abuse; median ICU length of stay was 41 (15 to 127) days and they were ventilated for 41 (12 to 114) days. Due to severe critical illness, target RASS was -4 for all patients, most of which were also paralysed. Isoflurane was administered in nine patients because of a high level of common sedative drugs, in five patients due to the use of two or more hypnotic drugs and in one patient because of hypertrigliceridemia. Isoflurane administration lasted 5.6 ± 1.8 days. During isoflurane administration no alteration in renal function or hemodynamic instability was recorded. After the isoflurane washout period we observed a reduction in sedative drug dosage in 10 patients while two patients were quickly weaned from mechanical ventilation and the target RASS raised to 0. In two patients isoflurane was precautionarily interrupted because of concomitant alteration of liver function and suspected seizures respectively.

## Conclusion

Inhaled anesthetics could be successfully used in the ICU especially in case of an inadequate sedation plan; for example, in patients with a history of drug abuse or young severe ARDS patients that required deep sedation and paralysis for a long period.

